# Reticulin‐Free Quantitation of Bone Marrow Fibrosis in MPNs: Utility and Applications

**DOI:** 10.1002/jha2.70005

**Published:** 2025-02-27

**Authors:** Hosuk Ryou, Emily Thomas, Marta Wojciechowska, Laura Harding, Ka Ho Tam, Ruoyu Wang, Xuezi Hu, Jens Rittscher, Rosalin Cooper, Daniel Royston

**Affiliations:** ^1^ Nuffield Division of Clinical Laboratory Sciences (NDCLS), Radcliffe Department of Medicine University of Oxford Oxford UK; ^2^ Big Data Institute/Li Ka Shing Centre for Health Information and Discovery University of Oxford Oxford UK; ^3^ Nuffield Department of Medicine University of Oxford Oxford UK; ^4^ Department of Cellular Pathology Oxford University Hospitals NHS Foundation Trust Oxford UK; ^5^ Ground Truth Labs, Ltd. Oxford UK; ^6^ Department of Engineering Science, Institute of Biomedical Engineering (IBME) University of Oxford Oxford UK; ^7^ Oxford NIHR Biomedical Research Centre Oxford University Hospitals NHS Foundation Trust Oxford UK; ^8^ Ludwig Institute for Cancer Research University of Oxford Oxford UK

**Keywords:** bone marrow pathology, diagnostic haematology, haematological malignancy, marrow fibrosis, myeloproliferative disease, machine learning

## Abstract

**Background:**

Automated quantitation of marrow fibrosis promises to improve fibrosis assessment in myeloproliferative neoplasms (MPNs). However, analysis of reticulin‐stained images is complicated by technical challenges within laboratories and variability between institutions.

**Methods:**

We have developed a machine learning model that can quantitatively assess fibrosis directly from H&E‐stained bone marrow trephine tissue sections.

**Results:**

Our haematoxylin and eosin (H&E)‐based fibrosis quantitation model demonstrates comparable performance to an existing reticulin‐stained model (Continuous Indexing of Fibrosis [CIF]) while benefitting from the improved tissue retention and staining characteristics of H&E‐stained sections.

**Conclusions:**

H&E‐derived quantitative marrow fibrosis has potential to augment routine practice and clinical trials while supporting the emerging field of spatial multi‐omic analysis.

## 1 Introduction

The assessment of fibrosis is a key component of bone marrow trephine (BMT) reporting in patients investigated for a myeloproliferative neoplasm (MPN) [1, 2]. Clinical diagnostic laboratories employ standardised silver impregnation techniques to highlight reticulin fibres (comprising type III collagen) that form a delicate network of thin, linear structures with variable condensation around bone and blood vessels within the normal marrow [3]. The pathological fibrosis characteristic of some MPNs at diagnosis or during disease progression reflects an increase in reticulin deposition, along with other collagen subtypes that include type 1 [4]. These features are incorporated into current European consensus criteria, but their application is subjective, only semiquantitative, and does not fully capture sample fibrosis heterogeneity [5, 6, 7]. We previously applied machine learning approaches to improve the detection and quantitation of marrow fibrosis in MPN using reticulin‐stained sections and demonstrated potential utility in the context of clinical trials [8, 9].

Notwithstanding advances in the quantitation of fibrosis using machine learning, challenges remain in generating consistently high quality reticulin‐stained sections suitable for manual or automated analysis. Silver impregnation techniques can be significantly affected by variations in the processing of BMT samples or the preparation of slides prior to staining [10, 11]. By contrast, most diagnostic laboratories are capable of consistently generating high quality haematoxylin and eosin (H&E)‐stained tissue sections. This reflects the relative simplicity of this stain and its ubiquitous application in diagnostic pathology, with laboratory staff and pathologists attuned to variations in quality control (QC) of H&E‐stained slides. In response, we sought to establish a reticulin‐free model of fibrosis quantitation derived directly from routine H&E‐stained slides.

## 2 Methods

As a first step, we generated H&E and reticulin whole slide images (WSIs) from identical tissue sections (*n* = 39) that spanned WHO MF grades 0–3. This was achieved using a stain–destain technique in which BMT sections were initially H&E‐stained on an automated Tissue‐TEK Prisma®, prior to coverslipping on a Tissue‐TEK® Glas™. After scanning (Philips ultra versatile scanner [UVS] L60/40×/NDPI), slides were placed into xylene to remove the pertex mountant before removal of the glass coverslip. The H&E‐stained tissue sections were then rehydrated in water prior to reticulin staining using a Gordon and Sweet's protocol, before being re‐coverslipped and scanned again at 40×.

**FIGURE 1:**
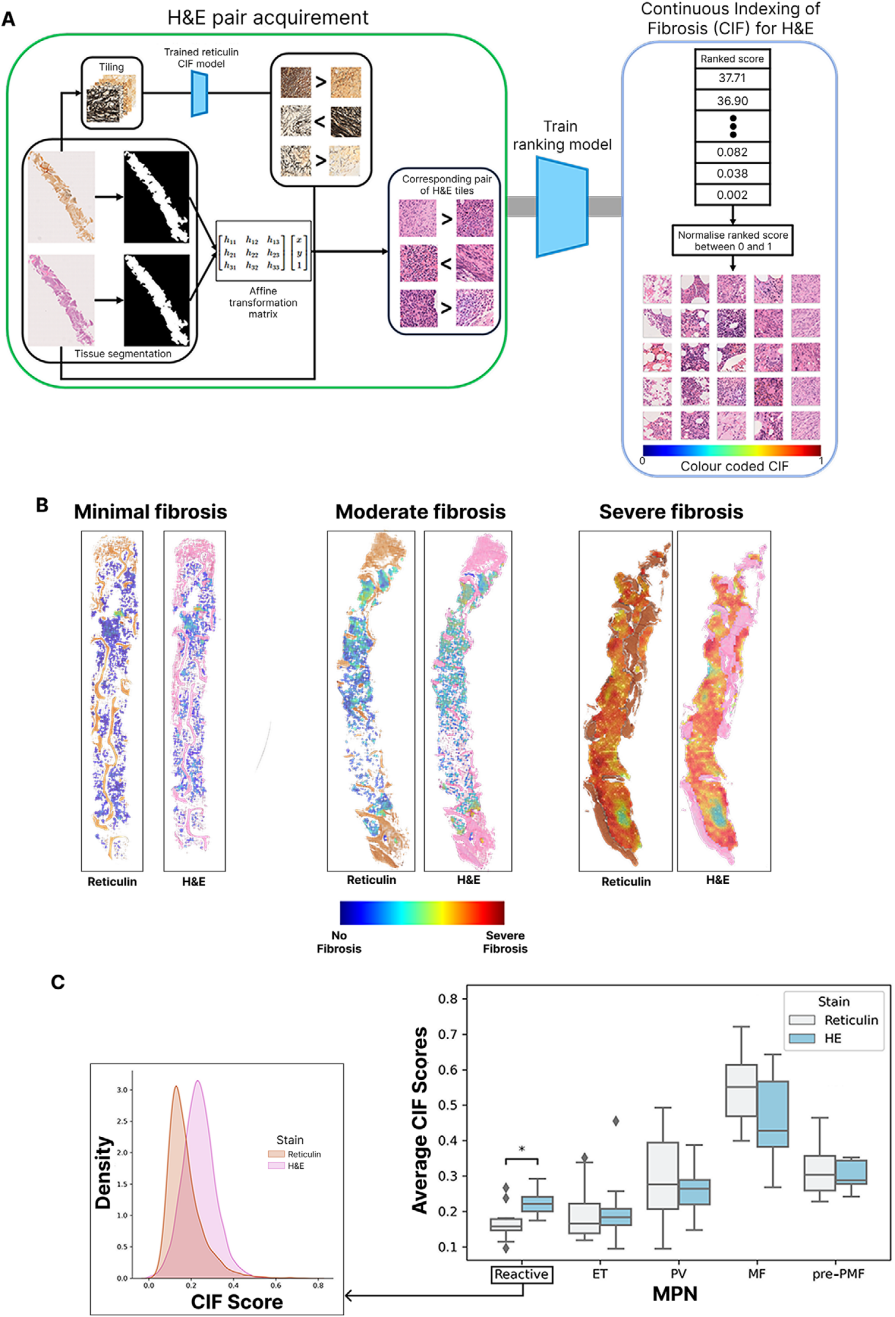
Overview and performance of the haematoxylin and eosin (H&E)‐derived Continuous Indexing of Fibrosis (CIF) model. (A) Schematic of the computational steps for the estimation of fibrosis using H&E‐stained bone marrow trephine (BMT) images. Reticulin image tiles were extracted from the whole slide images (WSIs) prepared during the stain–destain protocol (H&E followed by reticulin) and ranked based on CIF score, as described previously [8]. Corresponding H&E tiles were then aligned to the ranked reticulin tiles using an affine transformation matrix. A ranking convolutional neural network (CNN) model was trained on these CIF ranked H&E tiles to generate the H&E‐derived CIF model. (B) Examples of paired reticulin‐ and H&E‐derived CIF model outputs represented as false‐coloured heatmaps, including samples with minimal, moderate and severe fibrosis. (C) Boxplots comparing the reticulin and H&E‐derived CIF scores for reactive/normal marrow samples and each myeloproliferative neoplasm (MPN) subtype. The distribution of tile scores from both CIF prediction models for the reactive/normal samples is shown below. Results with *p* < 0.05 were considered statistically significant and are indicated by an asterisk.

For model training, tiles of 512 × 512 pixels (113 µm × 113 µm) were extracted from the reticulin WSIs within the tissue mask acquired by PathProfiler [12]. Continuous Indexing of Fibrosis (CIF) scores were predicted for these extracted tiles using the quantitative reticulin CIF model, as described previously [8]. An affine transformation matrix, derived from the segmented tissue masks of reticulin and H&E, was then applied to align reticulin tissue coordinates with the corresponding H&E images to acquire matched reticulin and H&E tiles. The dataset was divided into training (30 samples: 9252 tile pairs) and validation sets (nine samples: 1911 tile pairs) for H&E‐derived CIF model generation. As detailed previously, we employed a ranking convolutional neural network (ranking‐CNN) to capture the continuous spectrum of fibrosis severity from the H&E images (Figure 1A) [8, 9]. To address non‐uniform staining, we employed channel‐wise histogram equalisation as a pre‐processing step for input images [13]. All BMT tissue slides utilised in this study were fixed in 10% neutral buffered formalin prior to decalcification in 10% ethylenediaminetetraacetic acid (EDTA; 48 h) and cut at 4 µm.

## 3 Results and Discussion

The H&E‐derived CIF model achieved a prediction accuracy of 0.933, with the average CIF score difference between the models being small (0.075 ± 0.049). The average CIF scores from the reticulin‐ and H&E‐derived models for each MPN subtype were compared, with similar scores seen in cases of essential thrombocythemia (ET), polycythaemia vera (PV), myelofibrosis (MF) and pre‐fibrotic primary myelofibrosis (pre‐PMF; Figure 1C). However, it appeared to somewhat overestimate the CIF score of reactive samples (reticulin: 0.167, H&E: 0.222, *p*‐value = 2.554E−02), with an increase in estimated scores across the range of analysed H&E tiles suggesting this was not the result of any specific H&E feature. Importantly, the CIF maps generated from paired reticulin and H&E images appeared similar upon manual review, with retained detection of scattered foci of fibrosis within H&E‐derived CIF maps from heterogeneously fibrotic samples (Figure 1B).

**FIGURE 2:**
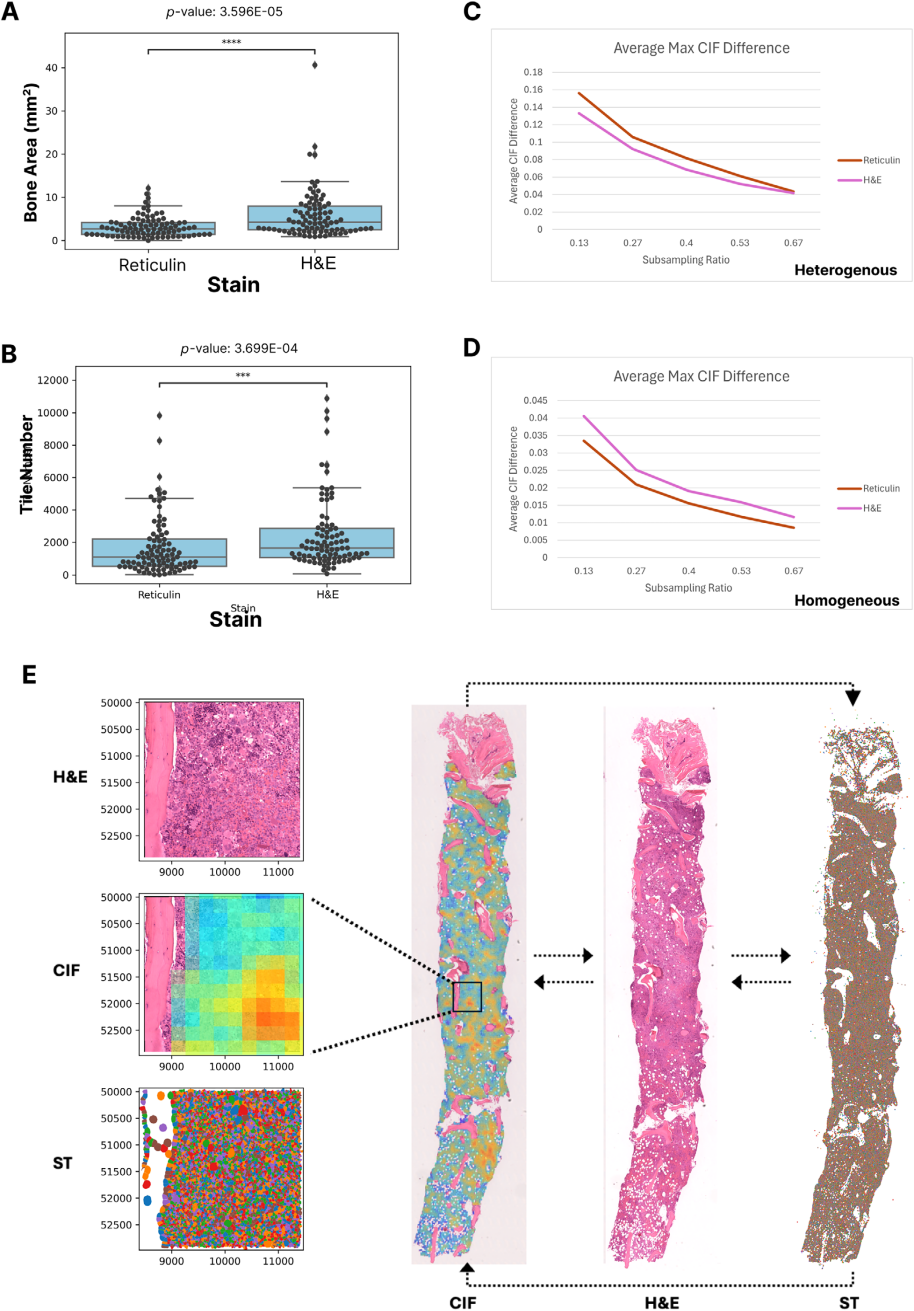
Quality control assessment of the reticulin and haematoxylin and eosin (H&E)‐derived Continuous Indexing of Fibrosis (CIF) models and application to spatial multi‐omic experiments. (A) Boxplots comparing the analysable bone surface area in paired reticulin‐ and H&E‐stained whole slide images (WSIs). (B) Boxplots comparing the total analysable tile number in paired reticulin‐ and H&E‐stained WSIs. Results with *p* < 0.05 were considered statistically significant. Influence of tissue subsampling on estimated sample CIF using the reticulin and H&E‐derived models for samples with ‘heterogeneous’ (C) and ‘homogenous’ (D) patterns of fibrosis, defined by the presence or absence of at least one smallest subsampling with a CIF prediction that deviated by > 0.1 from the whole sample score. (E) Generation of an H&E‐derived CIF heatmap enables precise correlation of fibrosis with cellular morphology/topology and spatial transcriptomic (ST) data. This can be derived from a single 4 um tissue section, without the need for coarse image registration from a sequential reticulin‐stained tissue section.

Next, we sought to explore the extent to which CIF maps generated from H&E‐stained slides may offer practical advantages over those derived from reticulin‐stained sections, with emphasis on staining quality and tissue integrity. For this purpose, we used a total of 89 diagnostic BMT samples in which sequential H&E‐ and reticulin‐stained sections were available (reactive/normal: *n* = 12, ET: *n* = 33, PV: *n* = 17, MF: *n* = 22, pre‐PMF: *n* = 5). Three sets of QC analysis were performed on the H&E and reticulin images: stain intensity variation; bone area preservation; and analysable intertrabecular area preservation. To assess stain intensity variation, we extracted the dominant colour from each sample image and converted them from RGB (red, green and blue) to CIELAB (Commission Internationale de l'Eclairage [CIE] Lab*), a colour space more suitable for comparison as it better approximates human vision [14]. This revealed significantly greater variance in the colour distribution for reticulin images when compared to H&E, as shown in both RGB and LAB (Supporting Information Figure 1). We then examined preservation of bone tissue by applying our previously developed bone segmentation model to WSIs [8]. This revealed that reticulin images contained significantly less intact bone tissue (average 2.717 mm^2^ per slide) when compared to H&E (Figure 2A). While not central to reticulin interpretation, loss of bone has potential to remove attached peri‐trabecular areas that could influence fibrosis assessment. To evaluate the total analysable area of the intertrabecular space for each sample, an experienced haematopathologist manually annotated intact intertrabecular spaces. Analysable tissue tiles that met our previously defined criteria (fat < 50%; bone or bone fragments < 1%) were then extracted. The average tile number of analysable intertrabecular tissue was seen to be significantly lower in reticulin images than the corresponding H&E (reticulin: 1720.546 ± 1789.021, H&E: 2476.652 ± 2207.423; Figure 2B).

A recognised concern when evaluating bone marrow fibrosis, either by manual MF grade or automated CIF score, is appropriately defining biopsy adequacy and estimating the influence of sample size. To interrogate this and compare our reticulin and H&E‐derived models, we subsampled the analysable tissue areas within each sample WSI to determine the impact on estimated sample CIF. Five sets of subsampling experiments were performed on each slide, with subsampled areas representing 2/15, 4/15, 6/15, 8/15 or 10/15 of the total analysable areas. For each subsampling, we randomly selected coordinates within the generated CIF map as the centres of subsampled regions, with expansion around this centre to the desired surface area ratio. Using the smallest subsampling region (2/15), we identified those cases in which any subsampled area generated a CIF prediction that deviated by > 0.1 from the estimated whole sample CIF score. Samples with at least one such region were subsequently classified as ‘heterogeneous’ for fibrosis (reticulin‐model: *n* = 25, H&E‐model: *n* = 21), with the remaining samples considered ‘homogeneous’ for fibrosis (reticulin‐model: *n* = 25, H&E‐model: *n* = 21). We then calculated the average maximum CIF difference between subsampled areas and the whole sample for both groups, comparing trends across each subsampling ratio (Figure 2C,D). As expected, for both the reticulin and H&E‐derived CIF models there was a greater difference between subsampled and whole sample CIF with reduced subsampled area, indicating a trend towards reduced accuracy in whole sample CIF prediction. There was only a minor (likely inconsequential) difference between models in samples defined as demonstrating ‘homogenous’ fibrosis (reticulin: 0.03–0.01, H&E: 0.04–0.01). However, within ‘heterogenous’ fibrosis samples there was a more striking decline in whole sample CIF prediction accuracy upon increased subsampling (reticulin: 0.16–0.04, H&E: 0.13–0.04), with the H&E‐derived model comparing favourably to the reticulin‐derived model.

In summary, we have developed a quantitative model of bone marrow fibrosis estimation (CIF) from H&E‐stained slides that benefits from improved tissue retention and staining characteristics when compared to reticulin‐stained images. The generation of such a quantitative output bypasses the subjective nature of bone marrow fibrosis assessment and captures the spectrum of fibrosis severity within and between manually determined WHO MF grades [8, 9]. This has potential to complement conventional marrow fibrosis assessment by pathologists. We consider the development of an H&E‐derived model to offer certain advantages in routine clinical practice and to have significant utility for researchers. For pathology laboratories within low‐ and middle‐income healthcare systems, the challenges associated with generating high‐quality reticulin stains could be overcome by extracting fibrosis estimation from an H&E slide that also provides the core diagnostic information required for BMT interpretation. Where reticulin‐stained sections are generated, an H&E‐derived CIF model has potential to support fibrosis estimation when reticulin images are of poor quality and simultaneously provide automated QC. The ability to quantitatively extract fibrosis severity from an H&E image also has significant potential within clinical trials of MPN in which study protocols frequently include the evaluation of fibrosis as a measure of disease modification/therapeutic response. The impact of variable reticulin stain quality or availability across participating laboratories in multi‐centre studies could be significantly offset by the generation of H&E‐derived CIF estimation, thereby enhancing the value of trial curated BMT WSI images for future image‐analysis studies. Such approaches will require validation studies across multiple diagnostic centres and integration into routine digital workflows. However, given the relative simplicity of generating consistently high quality H&E‐stained slides, we consider this approach highly suitable for such large scale validation studies. Finally, the advent of advanced whole slide multi‐omic approaches on routine diagnostic samples allows researchers to integrate multi‐modal advanced imaging techniques on single‐slide tissue samples. Indeed, we have recently combined H&E‐derived CIF estimation with whole slide spatial transcriptomics (ST) to interrogate the microenvironmental perturbations induced by MPNs and their relationship to marrow fibrosis [15]. Crucially, this offers increased precision by avoiding the need for inferential reasoning from a sequential reticulin‐stained section (Figure 2E).

## Author Contributions

**Hosuk Ryou**: study design, data generation, analysis, generating figures, reviewing and editing. **Emily Thomas**: generating figures, reviewing and editing. **Marta Wojciechowska**: analysis, reviewing and editing. **Laura Harding**: generating data, reviewing and editing. **Ka Ho Tam**: analysis, reviewing and editing. **Ruoyu Wang**: analysis, reviewing and editing. **Xuezi Hu**: analysis, reviewing and editing. **Jens Rittscher**: study design, reviewing and editing. **Rosalin Cooper**: analysis, generating figures, reviewing and editing. **Daniel Royston**: conceptualisation, resources, analysis, reviewing and editing. All the authors read and approved the final manuscript.

## Ethics Statement

This work was conducted as part of the INForMeD study (INvestigating the genetic and cellular basis of sporadic and Familial Myeloid Disorders; IRAS ID: 199833; REC reference: 16/LO/1376).

## Consent

All patients provided written informed consent.

## Conflicts of Interest

Ka Ho Tam, Xuezi Hu and Ruoyu Wang are employees of Ground Truth Labs Ltd. Jens Rittscher is a co‐founder of Ground Truth Labs and holds equity. Daniel Royston and Rosalin Cooper provide consulting services to Ground Truth Labs Ltd. The remaining authors declare no conflicts of interest.

## Clinical Trial Registration

The authors have confirmed clinical trial registration is not needed for this submission.

## Permission to Reproduce Material From Other Sources

Not applicable.

## Supporting information



Supporting Information

## Data Availability

The data that support the findings of this study are available from the corresponding author, DR, upon reasonable request.
