# Supplementary material for: Reticulin‐Free Quantitation of Bone Marrow Fibrosis in MPNs: Utility and Applications
Source: EJHaem. 2025 Feb 27;6(2):e70005. doi: 10.1002/jha2.70005 (PMC11866042; doi:10.1002/jha2.70005)
Supplement: Supplementary file 1 — Supporting Information [file JHA2-6-e70005-s001.docx]

**Supplementary Figure**


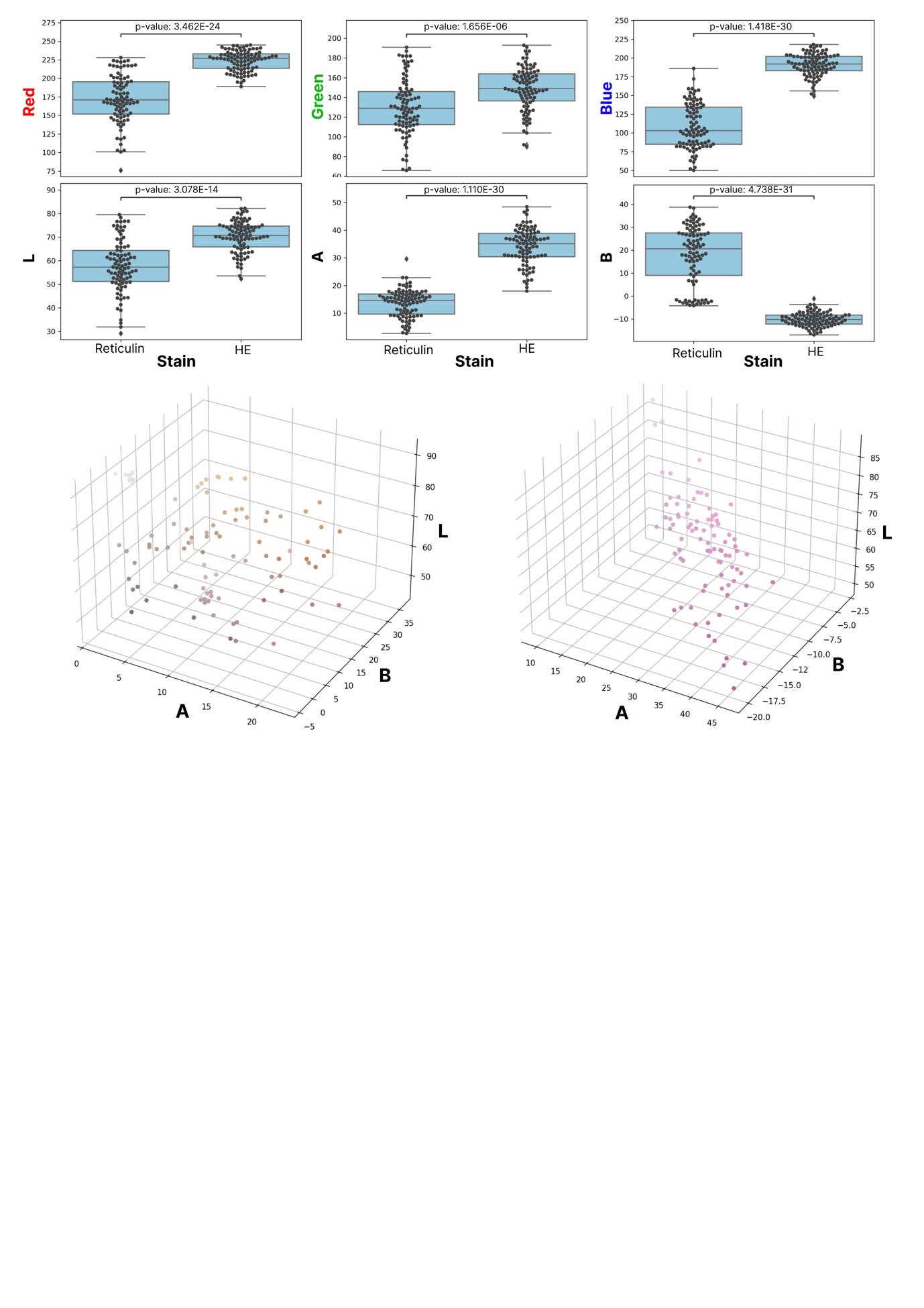


Supplementary Figure 1. **Stain intensity variation between the reticulin and H&E-derived CIF models for RGB (red, green and blue) and (CIE) Lab* shown as boxplots and combined 3D plots**. L* indicates lightness, A* is the red/green coordinate, and B* is the yellow/blue coordinate.
